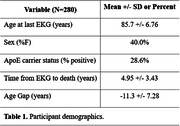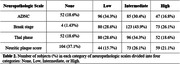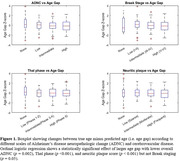# Older predicted age using AI EKG‐based age prediction is associated with higher Alzheimer’s disease neuropathologic change

**DOI:** 10.1002/alz.088037

**Published:** 2025-01-09

**Authors:** Camilo Bermudez, Jeremy A. Syrjanen, Zachi I Attia, Paul A Friedman, Francisco Lopez‐Jimenez, Mingzhao Hu, Ronald C. Petersen, David S. Knopman, Vijay K. Ramanan, Clifford R. Jack, Aivi T. Nguyen, Ross R. Reichard, Naomi Kouri, Christina M. Moloney, Alicia Algeciras‐Schimnich, Melissa E. Murray, Michelle M. Mielke, Prashanthi Vemuri, Peter A Noseworthy, Jonathan Graff‐Radford

**Affiliations:** ^1^ Mayo Clinic, Rochester, MN USA; ^2^ Department of Quantitative Health Sciences, Mayo Clinic, Rochester, MN USA; ^3^ Department of Neurology, Mayo Clinic, Rochester, MN USA; ^4^ Department of Laboratory Medicine and Pathology, Mayo Clinic, Rochester, MN USA; ^5^ Mayo Clinic, Jacksonville, FL USA; ^6^ Department of Neuroscience, Mayo Clinic, Jacksonville, FL USA; ^7^ Wake Forest University School of Medicine, Winston‐Salem, NC USA

## Abstract

**Background:**

There is increasing need for noninvasive biomarkers of Alzheimer’s Disease (AD) neuropathologic change for early detection and intervention through risk‐factor modification and disease‐modifying therapies. One such biomarker is the prediction of chronological age from routine clinical tests such as an electrocardiogram (EKG) to discriminate between observed biological age from chronological age for healthy aging. Deviation of true age from predicted age has been associated with heart failure, hypertension, and coronary heart disease. However, the association between EKG‐based age and underlying neuropathology has not been studied.

**Method:**

We used a cohort of 336 participants from the Mayo Clinic Study of Aging, a population‐based prospective study of residents in Olmsted County, Minnesota, with antemortem EKG and post‐mortem brain autopsy (Table 1 & 2). Age was predicted from EKG using a validated convolutional neural network algorithm. We used ordinal logistic regression to identify associations between lower neuropathologic scores of AD (Thal phase, neuritic plaque score, Braak stage, and overall AD neuropathologic change) and the Age‐Gap, defined as EKG‐predicted age minus chronological age. Results were controlled for true age, sex, and APOE e4 carrier status. Neuropathologic scales were grouped into none, low, intermediate, and high categories. Observations were weighted by the inverse of years between last EKG and time of death to account for the time difference between acquisition of EKG and autopsy.

**Result:**

We found that an increasing Age‐Gap (i.e. older predicted age) was inversely associated with lower overall AD neuropathologic change (OR 0.93 [0.89, 0.97], p = 0.002). Investigation of the components of AD neuropathologic change showed that an increasing Age‐Gap was also inversely associated with lower amyloid metrics such as Thal phase (OR 0.85 [0.80, 0.89], p < 0.001) and neuritic plaque scores (OR 0.91 [0.87, 0.96], p < 0.001), but not tau metrics like Braak staging (OR 1.05 [1.00, 1.10], p = 0.05).

**Conclusion:**

In this work, we show that older predicted age on EKG, a noninvasive biomarker associated with vascular and biological aging, is associated with higher amyloid burden and neuropathologic change of AD.